# Perceptions and experiences of diploma nursing students on clinical learning. A descriptive qualitative study in Tanzania

**DOI:** 10.1186/s12912-023-01362-1

**Published:** 2023-06-30

**Authors:** Angela Jacob, Saada Seif, Yuda Munyaw

**Affiliations:** 1 Department of Nursing Management and Education, School of Nursing and Public Health, University of Dodoma, Post Office Box 395, Dodoma, Tanzania; 2 Haydom Lutheran Hospital, Post Office Box 9000, Haydom, Mbulu Tanzania

**Keywords:** Clinical learning experience, Clinical learning perception, Learning environment, Diploma nursing students, Clinical teaching

## Abstract

**Background:**

The quality of nursing education depends largely on the experience student receive in the clinical environment. The learning environment is complex with factors that may positively or negatively influence students learning. The current study aimed to explore the experiences and perceptions of diploma nursing students toward their clinical learning in Dodoma-Tanzania.

**Methods:**

A qualitative descriptive study design was employed. The study was conducted in four nursing schools involving 32 nursing students who were purposively selected. Data was collected using focus-group discussions and analyzed using thematic analysis.

**Results:**

Three main themes emerged during the discussions: experience on personal and technical support for clinical learning, the importance of the clinical environment in clinical learning, and insufficient clinical educational planning. The majority of students had negative experiences including poor clinical supervision, lack of equipment, congestion of students, and inability to meet clinical objectives. Few students had positive experiences related to exposure to a real clinical environment and great support from staff nurses.

**Conclusion:**

Students had mixed experiences, both positive and negative on their clinical learning. The majority of students had negative experiences. This may have a serious impact on the student completing their education, the services they will offer to patients when employed, and nursing professional development.

## Background

Constructive clinical learning is a fundamental of nursing education [1], and through clinical practice students integrate cognitive, affective, and psychomotor skills to gain nursing clinical competencies [2]. Nursing competency is a core ability that is required to fulfill nursing responsibilities [3]. Nursing students are required to be placed in a standard clinical learning environment to enable them to acquire competence [4], as more emphasis is placed on practical training, and students are expected to gain competence in various nursing procedures before they can practice.

In Tanzania’s education system, students are enrolled in diploma schools and colleges after completing ordinary and/or advanced secondary schools. The diploma level is regarded as technical level regulated by statutory body National Council for Technical and Vocational Education and Training (NACTEVET) [5]. The diploma program takes three years, after which students graduate and enter the job market. According to Tanzania ordinary diploma curriculum for Nursing and midwifery, clinical practices takes approximately 570 to 670 h for one semester and 228 h for classrooms studies [6]. Based on the curriculum, all institutions under NACTEVET employs clinical placement which possess many hours as compared to classrooms’ hours to implement competence-based curriculum.

A standard clinical learning environment is defined as a complex atmosphere that includes staff, patients, and nurse educators that impacts nursing students’ careers by either enhancing or hindering their performance [7]. Flott & Linden highlighted four quality attributes of a clinical learning environment which are physical environment, interpersonal and psychosocial aspects, organizational culture, and clinical teaching components [8]. There is a complex interaction between these factors in such a way that students learning may be positively or negatively affected.

Factors such as motivated nursing staff, positive and supportive ward climate, and positive relationships between students and nursing staff have been documented to positively influence clinical learning [9, 10]. It is further documented that rigid ward routines, lack of team spirit and commitment to teaching nursing students, low staff morale, and inadequate supervision of students have a negative influence on the quality of students learning [10].

Studies report variations in clinical education experiences among student nurses [11]. A study conducted in Ethiopia revealed un-conducive clinical learning where nursing students reported that nurse teachers are not usually accessible to the students most of the time, and a single individual is assigned to a large group of students, which results in minimal one-to-one practical learning and demonstration sessions [12].

Another study further documented that lack of staff, lack of equipment and medical supplies, and short time allocated to some of the specialized units negatively affected students learning [3]. Additionally, learning is further affected by traditional methods of teaching and an increase in student enrollment while the infrastructure and staff almost remain the same [13].

Some studies have also documented positive clinical learning experiences by nurse students. In one study, Cypriot nursing students showed satisfaction with consistent supervisory relationships along with the regularity of individual meetings, the nurse’s teacher’s presence and support, and the sense of “team spirit” in well-organized nursing care [14]. In another study, students reported a positive experience in the clinical learning environment supervision, a pedagogical atmosphere, and collaboration between nurse teachers and students [15]. Across the globe, as the literature review shows, clinical learning is researched in various nursing educational aspects with conflicting results. To the best of our knowledge, there are few studies that reflect nursing students’ clinical experiences within a Tanzanian context.

In recent years, there has been an increase in the number of diploma colleges and student enrollment in Tanzania to meet nursing staff demands [16]. The diploma nurses contribute to the majority of nursing staff in the country [17]. The cadre doesn’t pursue internship practice after graduation; hence, clinical learning during their training is the only way to develop competency in patient care.

Therefore, this study aims to explore the experiences and perceptions of diploma nursing students toward their clinical learning. The reflections obtained from students as true owners of the learning process might help to strengthen clinical teaching and improve nursing education programs.

## Methods

### Study design and setting

This study adopted a qualitative explorative descriptive design that allowed the understanding of students’ experiences and perceptions of clinical learning and describe them. The study was conducted in four nursing schools located in the Dodoma region of central Tanzania that offer diploma in nursing. The region has four nursing school institutions awarding the diploma and two awarding the undergraduate and postgraduate degree in nursing. Students from all institutions in the region practice their clinical skills in six district hospitals, one regional hospital and one zonal hospital, and in 39 health centers under government and private ownerships in Dodoma region.

### Study population and sampling

The study population was diploma nursing students recruited from four nursing institutions in Dodoma. There were a total of 453 diploma nursing students in all four institutions at the time of the study. This study included only students who were in their second or third year of study. The clinical placement of the diploma nursing students in these institutions starts when students are in their second semester of year one of study and continues up to their final third year of study. Thus, those who are in their second and third year of study would have spent more time in the clinical area to be able to explore their perceptions and experiences of clinical learning. A purposive sampling technique using a criterion strategy [18] was used to select study participants. The predetermined criteria included having completed at least one clinical rotation at the time of this study, be it in medical units, surgical, pediatric, obstetric, and labour units, theaters, emergency rooms, or outpatient units. A list of diploma nursing students was requested from the school by the principal investigator, and only those who met the inclusion criteria were selected to take part in the study. All the students who were invited to participate in the study agreed to do so. The study started with 8 participants in one focus group discussion (FGD). The analysis started immediately after each discussion, so more recruitment was carried out until no new information was obtained. A code saturation was used, and the saturation was reached after four FGDs with 32 participants. Among the 32 participants, nineteen were female and thirteen were male. The participant’s ages ranged from 19 to 25 years old, twenty were in their third year of study and twelve were in their second year of study (Table 1).

**Table 1:** Demographic information of participants (Number = 32)

Characteristics	Number
Age	
< 20	9
20–25	18
> 25	5
**Year of Study**	
Year 2	12
Year 3	20
**Gender**	
Female	19
Male	13
**Institution of study**	
Institution1	8
Institution2	8
Institution 3	8
Institution 4	8

### Data collection and analysis

The data was collected using FGD from April to June 2022 by the principal investigator and two trained research assistants who are nurses by profession. The principal investigator is working as a nurse tutor in a nursing school that was not involved in this study. She is aware of the students’ curriculum. The discussion was carried out in a quiet place in the nursing school institution at a date, time, and venue that were convenient to the informants. Each FGD took about 45 to 60 minutes to complete, and they were moderated in Swahili, the national language of the country that is spoken by the majority.

The FGD was guided by the semi-structured interview guide adapted from Mbakaya et al. [19] to ensure the discussion was within the intended topics and discussed uniformly. The discussion is based on the following themes: (i) experiences of clinical supervision; (ii) experience in integrating theory to practice; (iii) expectations of learning from teachers, nurses, and the environment; (iv) teaching approaches used; (v) working relationships in the clinical area; (vi) nature of ward activities involved; (vii) meeting clinical objectives; and (viii) challenges encountered. During the interviews, probe and follow-up questions were posed to gain detailed information. All discussions were audio recorded with the permission of participants. During the discussion, member checking was performed to confirm the understanding of the idea explained.

Data were analyzed manually using thematic analysis, which started immediately after each discussion. Six steps as described by Braun & Clarke for thematic analysis were used to guide the manual interpretation of the data [20]. The process started with the familiarization of the data whereby the recorded FGD were transcribed verbatim in Swahili by the principal investigator, and the transcripts were read several times by all team members while actively listening to the recorded data to understand the contexts and to note any areas that were incorrectly transcribed and to correct them with the help of field notes. This was followed by the extraction of meaning units from the transcripts. The meaning units were condensed by shortening the original text while maintaining the central meaning. The condensed versions were later assigned codes. A set of codes was constructed to describe groups of categories with comparable meanings. The groups of categories were refined, and themes emerged. The research team reviewed all codes and themes emerging from the transcripts. Any discrepancies regarding data analysis were discussed among the researchers, and agreement was reached through the majority rule. In the final phase, the researchers wrote a detailed account of the thematic analysis findings. The description of the findings reported using narrative descriptions, and quotes were translated into English. The principal investigator always cross-referenced between Swahili and English to ensure that the meaning units, codes, and themes of the two languages were congruous. Peer debriefing was carried out, whereby two experts in qualitative study from two universities were requested to review the transcripts, methodology, and findings. Any element of incongruence and subjectivity was communicated, the transcripts and audio recordings were revisited to ensure the report accurately portrays the informant’s responses. Three main themes emerged from the generated codes: (1) Experience on personal and technical support, (2) Importance of clinical environment for clinical learning and (3) Insufficient clinical education planning (Table 2).

**Table 2:** Generated codes and themes on the description of perception and experiences of students’ clinical learning

Meaningfully sentence	Codes	Subthemes	Themes
*…. From my experience, teachers come around the clinical area, but it is not that they are coming to teach us, no teachers are passing by to check if students today showed up in the ward or not?*	Nurse tutors rarely come to teach students	Feel abandoned	Experience on personal and technical support for clinical learning
	Unfriendly speech from clinical instructors		
	Shortage of clinical instructors	Lack of supervision support	
	Busy schedule of clinical instructors		
	Other teaching methods are not commonly used	Clinical teaching expectations disappointment	
	Expect to practice under supervision of school teachers and not only clinical instructors		
*The experience I had at the clinic was great we learned a lot from the staff…….*	Great experience from some staff	Staff nurses are supportive	Importance of clinical environment for clinical learning
	Some staff provide assistance to perform procedure and correct us		
	Exposure to real practising environment	Perfect place for learning	
	Ward activities are well structured and beneficial		
	A large group of nursing students compared to the available space	Inadequate requirement for learning	
	Not able to see the demonstrated procedure		
	A limited number of patients		
	Failure to do procedure step by step due to lack of equipment’s		
	Staff uses experience than standard operating procedures	Theory-practice gap	
	Applying theory to practices sometimes brings confusion		
	Lack of correct information		
	Missing ward round	Poor clinical learning schedule	Insufficient clinical educational planning
	Missing morning report		
It is very difficult to catch the hands-on skills because in one-unit I stayed for only one week ….	Early shifting from one unit to another	Inadequate time for practice	
	Short clinical time		
	Unclear job assignment	Unable to meet clinical own objective	
	Un-necessary clinical task		

### Rigour of the study

The trustworthiness of the data was assessed by its credibility, dependability, conformability, and transferability throughout the study as stated by Barusch et al., [21, 22]. The credibility that entails trusting the findings of the study [22] was ensured by member checking during data collection to confirm the understanding of the idea and to highlight the areas that were missed or misunderstood. Peer debriefing was also carried out, whereby independent experts were requested to review the transcripts, methodology, and findings. The dependability, which entails the extent to which similar results will be produced if the study is repeated in the same context using the same population and methods, was accomplished by adapting the data collection tool, having it reviewed by experts in nursing education and piloting it, and having experts in qualitative studies from the University of Dodoma, Tanzania, review the analysis process. The thick description of study participants and details of the steps of how this research was carried out were given to ensure transferability of the findings, which entails the applicability of findings to other settings. And the confirmability, which entails the extent to which the researcher’s bias, motivation, or interest are controlled, thereby ensuring that the findings of the study portray the participants’ responses and are not colored by conscious or unconscious bias from the researcher, was achieved by a detailed description of how data were analyzed, starting from summarizing the content of each question we ask during the FGD, extracting the meaning units, condensing them, and how codes were assigned and combined to generate categories and themes.

### Ethical approval

Ethical clearance was obtained from the Institutional Review Board of the University of Dodoma (UDOM). Administrative permission was obtained from the Ministry of Health, training department and from the Principals of the respective schools. Written voluntary informed consent was obtained from each participant. Participants were assured their right to withdraw from the study at any time. The researchers were aware of the students’ vulnerable positions, especially as their role as students may have discouraged them from withdrawing. Therefore, before each FGD, the students were reminded that they would be asked about their personal experiences and perceptions about their clinical learning, thus giving them additional opportunities to assent to or withdraw from the study. To maintain confidentiality, students’ names were not used. The collected data forms were safely kept by the PI and shared only with other investigators in the study. None of the students suggested any discomfort during FGD, and none chose to withdraw.

## Findings

### Theme 1. Experience on personal and technical support

Clinical placement is the core component of nursing education. The clinical instructors, hospital staff and the academic faculty’s roles and responsibilities are to supervise clinical activities and to empower nursing students to acquire competency in providing quality and cost-effective care among patients [23]. The finding of this study revealed that students have mixed experiences, both positive and negative, on the type of personal and technical support they receive during their clinical placement. On the positive experience students praised staff nurses for their support and willingness to assist students to attain clinical competences, as one participated said:

*“So far, I have experienced many things. I’ve seen the staff cooperate with us, and if we make mistake, they correct us...”****(P28, fgd4).***

While on the negative experience, which took the large share of the discussion, students described their disappointment on the clinical supervision they expected to get from the clinical instructor and from their tutors. Their description is likened to feel abandoned. They described that they got difficulty in accessing the assigned clinical instructors as they are always too busy with their schedules, and do not have time to supervise and direct students in their practice. Moreover, their teachers from the school rarely visit them to teach the clinical skills. The following student’s statements reflect this claim:

*“In this rotation, I have been assigned to rotate in the labor ward, postnatal ward, and antenatal ward, but my clinical instructor has multiple responsibilities that resulted in inadequate follow-up or even teaching and evaluating the activities that I performed in the clinical settings“****(P7, fgd1).***

Students explained that their expectation was different from reality. The majority of the respondents expected to see their teachers frequently in the clinical area but in real sense tutors rarely visit them. One participant claimed:

“*I expected that when we were in the ward the teachers would continue to be with the students and continue supporting us, I expected teachers to come and guide us on how to perform the procedures. However, they rarely did come”****(P19, fgd3).***

### Theme 2. Importance of clinical environment for clinical learning

The clinical learning setting influences the realization of proven clinical learning outcomes and help in the advancement of efficient professional performances among nursing students [24]. During FGD, students showed great satisfaction to the exposure to real clinical environment where they can practice with real patients. They suggested that the clinical environment as a perfect place to learn the clinical skills. Practicing in real clinical environment helps to build more confidence and a sense of responsibility in caring for patients. Moreover, the well-organized ward activities provide positive desire to continue working in the clinical areas. One participant said:

*“…. Yes, the ward activities are well structured and benefited us because the task we provided are for learning, you find something you haven’t taught in class but in the ward the staff will guide and teach you…we learn through practice”****(P9, fgd2).***

However, participants raised concerns about the adequacy of clinical environment for their clinical learning. The discussion on this part dominated by the negative experiences of students in the clinical environment, the main challenges raised were the inadequate medical supplies both equipment and material like gloves, inadequate space compared to number of students, low number of patients to practice to compared to number of students, unsupportive staff, limitation in applying the theory learned to practice. On inadequate equipment one participant said:

*“There was lack of enough dressing kits and screens for maintaining patients’ privacy, so I was performing the procedure while other patients were observing”****(P17, fgd3).***

Participants further described that there is overcrowding of students from different colleges and different cadres. Sometimes this leads to one patient being attended by many students risking the patient’s confidentiality and privacy, patients became aggressive when we practice on them the same thing at short intervals so they feel tired and annoyed, as quoted below:

*“The challenge I see is that students are many if you look here in Dodoma there are many colleges and most of the students from those colleges, we attend clinical placement here in the X hospital...Imagine undergraduate (Bachelor of Nursing) students, diploma, certificate, clinical officers, medical students, and interns, all in the same ward…so it is hard for each student to have a patient to take care of and prepare a nursing care plan. And during ward rounds, only medical students were allowed to learn, we nursing students we just distribute the patient’s files and pushing a trolley for the round“****(P25, fgd4).***

On the challenge of unsupportive staff, participants reported that they face unfriendly learning environments such uncooperative staff using harsh language to students. Students explained that being shouted at or discouraging words make them feel bad as human beings, they elaborated more that it may be true that they are not competent but that’s not supposed to be the reason because they lose confidence to practice. Others stated that they need supportive direction from staff and good language that can promote their learning and reduce fear to practice, as quoted below:

*“For me, the experience was not good…There was a day we were in ward number 1 and then one of us was told to draw medicine from an ampoule, in the process of drawing medicine he made a little mistake…the nurses were quite harsh and spoke up until the patients looked at us…Making it difficult to feel comfortable practicing again. You just want the hours to go fast and the duty to end“****(P1, fgd1).***

Participants reported theory-practice gap as a barrier, the majority of the participants mentioned inconsistence of information, probability due to updated guideline, nurse’s shortcuts on performing procedures and lack of follow up of updates or sharing of information on updates among tutors and nurse/hospital management, one student said:

*“I see there is a difference between what we learned and what we encounter in the clinical area because there is a lack of resources, also in the ward nurses often does not follow the procedure. This makes it challenging for us to apply theory to practice“****(P4, fgd1).***

### Theme 3: Insufficient clinical education planning

The ability of nursing practices was educated and supervised in clinical education through the implementation of clear and concise job allocation and planned clinical objectives among nursing students. Students preferred completion of their plan before the end of the shift. The majority of participants explained their experience that completion of clinical objectives was a problem for most of them. Students discussed short placement stay in some wards and irregular rotation plan as the attribute that affects their clinical learning and teaching. Students discussed clinical learning schedule was too irregular that sometimes they took a long time in some wards while in other wards they took short time. They stated that the most difficult time was when they were approaching the examination period and being shifted to a new ward, as narrated below:

*“It is very difficult to learn hands-on skills in the clinical setting because of short clinical placement, for example in my recent clinical placement I was allocated to the labor ward and my task was to practice stages of labor and assessment of the pregnant women during labor but I didn’t fulfill the needs because I had a very limited time and my next roster wanted me to be in the surgical ward“****(P6, fgd1).***

Participants also raised concern about the unclear nursing roles they experienced in clinical areas. They found this confusion from other nurses and other medical staff. They complained of being assigned to other tasks not related to their profession like to take sample to laboratory, or to go to pharmacy or other units, and they remained with little time to complete their objectives. One participant said:

*“I was feeling uncomfortable to elaborated my gratitude to nurses because the majority of them were just sending us to take a blood sample and take medication and I was just getting confused due to running and unnecessary tasks that made me fail to evaluate the objectives that I performed“****(P13, fgd2).***

Another participant added on that:

*“For me, I don’t think if clinical environment meets my objective, like you can plan your objective that today I want to learn wound dressing but once you reach a clinical area you end up being on an errand, so the environment for me is not supportive”****(P25, fgd4.)***

## Discussion

In this study, we explored the experiences and perceptions of diploma nursing students toward their clinical learning. The findings show that students have mixed experiences, both positive in some aspects and negative in others. The positive experience needs to be cherished and maintained, but the negative experience may contribute to low performance and incompetence among nursing student graduates. Previous studies have shown that student learning depends on effective clinical education preparation and the establishment of a suitable learning environment [11].

Supportive supervision of students during clinical placements makes them competent and confident [25, 26]. In our study, large share of students’ statement indicated dissatisfaction with supervision during clinical placements. This concurs with the findings from other studies [19, 27, 28]. Student supervision in clinical settings is supposed to be conducted by nurse teachers from the school and clinical instructors from healthcare facilities. However, the shortage of nurse teachers and clinical instructors in low-resource settings is not uncommon [29, 30]. This makes students spend more time in clinical areas without getting any technical support from the nursing school or from the practicing hospital. Additionally, students view that a lack of supervision in clinical placements imposes fear and anxiety which in turn negatively impacts their learning experiences.

Students reported challenges that they come across in clinical settings such as a shortage of equipment for procedures, a large number of students, poor support from staff nurses, and a theory-practice gap. Other studies have also reported the challenges of inadequate equipment for practicing procedures [3, 19, 31]. A large number of students can give students the opportunity to share and learn from each other as they have different exposure of theory from different teaching institutions. However, this is only effective if there is adequate supervision from the school teachers or clinical instructor, who will plan, direct, and organize the learning activities including collaborative learning. Results from a systematic review [32] demonstrated that collaborative learning across disciplines can initiate team work in health care and develop the ability to be effective in learning and team work.

The large number of students is due to an increase in the number of nursing teaching institutions and the enrollment of a large number of students in some parts of the country. However, the number of teaching hospitals has remained more or less the same. This contributes to the congestion of students at clinical sites, as described during FGDs. The finding is consistent with that of Jamshidi, who reported an increase in the number of health students enrolled, which leads to an increase in demand for clinical placement sites [33].

The consequences of students’ congestion and limited hands- on practice are incompetent nurses after graduation, which in turn risk the health of patients. One of the approaches employed in other settings to solve the problem of limited hands-on practice in clinical placement is to use simulation-based practice. A study conducted in Australia revealed that the demand for clinical placement is not more of a problem as they shifted to simulation-based training where the students practice using manikins [13]. There is a need to adopt simulation-based practices in the students’curriculum and training to strengthen their skills. However, the challenges in low resource settings in setting up simulation-based practice are apparent due to inadequate funds to support the establishment of the skills laboratory and inadequate trained personnel for setting up the scenarios and assessment using simulation.

The participants of this study further described that nurses are too busy with their work, while others are not willing to support or teach nursing students, claiming that they are too busy with their schedule. Similar findings were reported by Donough and Van der Heever, [19, 27] that clinical nurses were preoccupied with administrative tasks and patient care, which made them less supportive of students’ practical learning. As staff nurses have a crucial role in supervising, counseling, and evaluating students in the clinical setting, there is a need to bridge the gap by engaging them closely in students’ academic activities. One of the proposed ways to address this challenge is to set a working agreement between faculty, staff nurses, and hospital management by setting a contract for who should supervise the students and at what intervals, with signed agreements [34].

During FGDs, students explained the inconsistency between what they have been taught in class and what is practiced in the clinical setting. The explained reasons behind this include a lack of resources, which necessitates staff to improvise equipment. Additionally, there is a lack of updates on guidelines and standard operating procedures among staff. Other studies have also reported theory-practice gaps during clinical placements [35, 36].

The findings of this study bring attention to academic institutions and teaching hospitals. There is a need to consider and develop new ways to help students in the wards. The nursing schools have to consider giving teachers additional resources for clinical supervision. Hospitals should encourage staff nurses to maintain their professional integrity so that they can practice standard nursing care by following standard operating procedures and serving as role models for students. As clinical placements act as a major contributing factor to building professional identity, students should not be unnecessarily engaged in activities unrelated to the nursing profession.

### Strengths and limitations

To our best knowledge, this study is novel as it is the first to explore nursing students’ experiences regarding clinical learning in hospital context in Tanzania. The participants provided rich and different descriptions of their experiences which gave a broad insight of clinical learning. With ensured quality data collection and analysis, the study rigour is high and the results are credible. Despite these strengths, the findings may be limited to the students’ perspectives, however, transferrable to similar contexts.

## Conclusion

Students had mixed experiences on their clinical learning, both positive and negative. However, the majority had negative experiences. This may reflect poor quality of clinical teaching which in turn impairs quality of healthcare patients receive from nurses after graduation. It may also affect the nursing schools in future by failure to obtain competent personels/staff for faculty development.

## Data Availability

Data may be available on reasonable request from the corresponding author.

## Abbreviations

FGD: Focus Group Discussion
UDOM: University of Dodoma
NACTEVET: National Council for Technical and Vocational Education and Training
